# Internet use, cardiometabolic multimorbidity, and death in older adults: a multi-cohort study spanning developing and developed countries

**DOI:** 10.1186/s12992-023-00984-z

**Published:** 2023-11-06

**Authors:** Ziyang Ren, Shuangbo Xia, Jinfang Sun, Duoduo Wang, Yushan Du, Ning Li, Jufen Liu

**Affiliations:** 1 Institute of Reproductive and Child Health/National Health Commission Key Laboratory of Reproductive Health, Peking University, No 38 College Rd, Haidian District, Beijing, 100191 China; 2 Department of Epidemiology and Biostatistics, School of Public Health, Peking University, No 38 College Rd, Haidian District, Beijing, 100191 China; 3 Office of Epidemiology, Chinese Center for Disease Control and Prevention, Beijing, China; 4 Institute of Population Research, Peking University, No 38 College Rd, Haidian District, Beijing, 100191 China

**Keywords:** Internet use, Old adults, Cardiometabolic Disease, Death, Multi-cohort, Meta-analysis

## Abstract

**Background:**

Internet use is a double-edged sword for older adults’ health. Whether internet use can prevent cardiometabolic diseases and death in older adults remains controversial.

**Methods:**

Four cohorts across China, Mexico, the United States, and Europe were utilized. Internet use was defined using similar questions. Cardiometabolic diseases included diabetes, heart diseases, and stroke, with 2 or more denoting cardiometabolic multimorbidity. Depressive symptoms were assessed using the Center for Epidemiological Studies Depression Scale and Europe-depression scale. The competing risk analysis based on subdistribution hazard regression, random-effects meta-analysis, and mediation analysis were utilized.

**Results:**

A total of 104,422 older adults aged 50 or older were included. Internet users (vs. digital exclusion) were at lower risks of diabetes, stroke, and death, with pooled sHRs (95% CIs) of 0.83 (0.74–0.93), 0.81 (0.71–0.92), and 0.67 (0.52–0.86), respectively, which remained significant in sensitivity analyses. The inverse associations of internet use with new-onset cardiometabolic diseases and death were progressively significant in Mexico, China, the United States, and Europe. For instance, older internet users in Europe were at 14-30% lower cardiometabolic risks and 40% lower risk of death. These associations were partially mediated by reduced depressive symptoms and were more pronounced in those with high socioeconomic status and women. Furthermore, patients with prior cardiometabolic conditions were at about 30% lower risk of death if they used the internet, which was also mediated by reduced depressive symptoms. However, certain cardiometabolic hazards of internet use in those aged < 65 years, with low socioeconomic status, men, and single ones were also observed.

**Conclusion:**

Enhancing internet usage in older adults can reduce depressive symptoms and thus reduce the risks of cardiometabolic diseases and death. The balance of internet use, socioeconomic status, and health literacy should be considered when popularizing the internet in older adults.

**Supplementary Information:**

The online version contains supplementary material available at 10.1186/s12992-023-00984-z.

## Introduction

With population aging, the past three decades have witnessed a noticeable increase in mortality and years of life lost (YLLs) attributed to cardiometabolic diseases (CMDs) [1], rendering identifying protective factors to prevent CMDs at early stages and improving the prognosis for patients with CMDs of utmost importance.

Despite the rapid advances in internet technology and its widespread use in recent decades, internet usage among the elderly remains low, particularly in developing countries [2]. Recently, evidence has indicated that older internet users are less likely to experience mental health issues such as loneliness and physical disorders like functional limitations [2–4]. Internet use can also facilitate access to health information for the elderly, thus improving their health literacy [5]. Coupled with the massive demand for internet use brought about by the COVID-19 outbreak, increasing internet usage among the elderly has become a priority [6–8].

Emerging evidence suggests that older adults who use the internet are at a lower risk of developing depressive symptoms and are more likely to foster healthy lifestyles, which are significantly associated with CMDs or cardiometabolic multimorbidity (CMM) [9–12]. Internet-based interventions are also advocated for patients with CMDs to improve their prognosis [13, 14]. However, recent research has indicated that excessive internet use among older adults may contribute to an increased risk of depressive symptoms [15], suggesting that internet addiction should also be a concern for the elderly. Accordingly, it is imperative to confirm whether internet use can indeed benefit the cardiometabolic risks in older adults and to identify potential effect modifiers to mitigate internet addiction. Utilizing data from multi-cohorts, including countries with different levels of development and sociocultural backgrounds, can help to explore this issue from a global and social epidemiology perspective. Nonetheless, limited related research has been conducted to date.

To bridge these research gaps, our study aimed to explore the associations of internet use among older adults with new-onset CMDs, CMM, and death, as well as the mediating role of depressive symptoms, based on cohorts from developing countries such as China and Mexico and developed countries including the United States and European countries, to obtain a wider global perspective.

## Methods

### Study design and population

Data were obtained from four international cohorts targeting middle-aged and older populations, namely, the China Health and Retirement Longitudinal Study (CHARLS) and Mexican Health and Aging Study (MHAS) from developing countries in the East and West, and the Health and Retirement Study (HRS) and Survey of Health, Ageing and Retirement in Europe (SHARE) from developed countries, which were designed to provide comparable results.

The CHARLS was approved by the Institutional Review Board and Ethics Review Committee of Peking University [16]; the MHAS was approved by the Institutional Review Boards and Ethics Committees of the University of Texas Medical Branch in the USA, the National Institute of Statistics and Geography (INEGI), and the National Institute of Public Health (INSP) in Mexico [17]; the HRS was approved by the institute for social research and survey research center of the University of Michigan [18]; and the SHARE was approved by the Ethics Committee of the University of Mannheim and the Ethics Committee of the Max Planck Society [19]. Written informed consent was obtained from all participants.

**Fig. 1:**
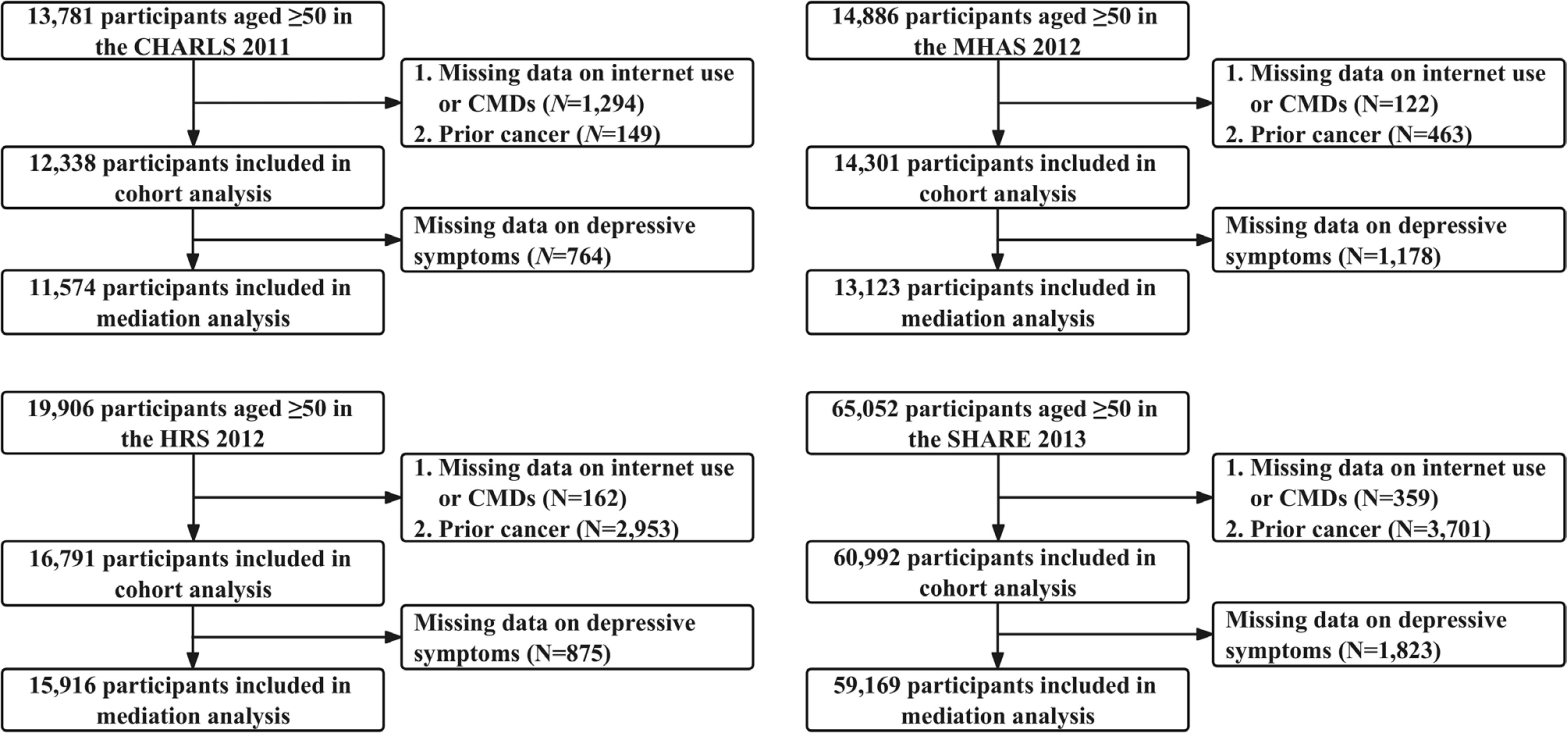
Flowchart of study. Notes: CHARLS, China Health and Retirement Longitudinal Study. MHAS, Mexican Health and Aging Study. HRS, Health and Retirement Study. SHARE, Survey of Health, Ageing and Retirement in Europe. CMDs, cardiometabolic diseases

In this study, the CHARLS 2011–2018, MHAS 2012–2021, HRS 2012–2020, and SHARE 2013–2020 were utilized (Fig. 1).

### Definition of internet use and digital exclusion

In the CHARLS, participants who reported using the internet within the last month were classified as internet users. For the MHAS, participants were considered internet users if they reported having internet access as part of their household electronics. In the HRS, participants were asked: ‘‘Do you regularly use the Internet (or the World Wide Web) for sending and receiving e-mail or for any other purpose, such as making purchases, searching for information, or making travel reservations?”. Those who answered yes were considered internet users. In the SHARE, participants were asked if they had used the internet within the past seven days, and those who answered yes were classified as internet users. Internet use among four cohorts was defined at baseline, and participants who did not meet these criteria were categorized as digital exclusion [2].

### Definition of CMDs and death

CMDs in the CHARLS, MHAS, HRS, and SHARE included diabetes, heart diseases, and stroke. Diabetes in the CHARLS was defined as fasting plasma glucose ≥ 7.0 mmol/L, and/or random plasma glucose ≥ 11.1 mmol/L, and/or HbA1c ≥ 6.5%, and/or with physician diagnosis or treatment, while that in the MHAS, HRS, and SHARE was defined using physician diagnosis or treatment given data limitations. Furthermore, heart diseases and stroke in these cohorts were defined by participants’ self-reported physician diagnoses. The presence of two or more CMDs in the same individual was classified as CMM.

Death was confirmed through proxy respondents, such as family members, household members, or neighbors.

### Measurement of depressive symptoms

Depressive symptoms were assessed at baseline in the four cohorts using the Center for Epidemiological Studies Depression Scale (CESD) or the Europe-depression (EURO-D) scale. Detailed information can be found in **eMethods in the Supplement**.

### Covariates

Information on age, sex, education, household income, marital status, smoking history, drinking history, and hypertension was collected through face-to-face interviews or telephone interviews at baseline. Education was classified as less than high school, high school, and college or above. Household income was divided into first, second, and third tertiles in sequence. Marital status included married/cohabiting and single. Smoking and drinking history were classified as current smoking/drinking or not. Hypertension was defined using participants’ self-reported physician diagnoses.

Body mass index (BMI) was measured by trained health workers in the CHARLS, MHAS, HRS, and SHARE at baseline. However, for HRS respondents who had missing data on their BMI, self-reported BMI values were utilized. Abnormal weight was defined as BMI ≥ 25 kg/m^2^.

### Statistical analysis

The baseline characteristics of included participants were described as medians with interquartile ranges (IQRs) for age given its skewed distribution, and frequency and per cent (%) for categorical variables. Missing data on covariates were multiply imputed using the ‘mice’ package in R.

The competing risk analysis based on subdistribution hazard regression was utilized to investigate the associations of internet use with new-onset diabetes, heart diseases, stroke, and CMM after excluding those with corresponding diseases at baseline and considering the competing risk of death in the CHARLS, MHAS, HRS, and SHARE, respectively. The proportional hazards assumption was verified using scaled Schoenfeld residual tests. All models were adjusted for age, sex, education, household income, marital status, current smoking, current drinking, abnormal weight, and hypertension.

Two sensitivity analyses were conducted: First, complete-case analysis after excluding those with missing data; Second, excluding new-onset CMDs and CMM within the next wave to account for potential latency time windows and avoid the potential reverse-causality bias.

The mediating role of depressive symptoms in the association of internet use with new-onset CMDs, CMM, and death in the CHARLS, MHAS, HRS, and SHARE was further investigated using the mediation analysis.

Furthermore, age (< 65, >=65)-, sex (men, women)-, education (less than high school, high school, college or above)-, household income (first, second, third tertiles)-, and marital status (married/cohabiting, single)-stratified associations of internet use with new-onset CMDs, CMM, and death in the CHARLS, MHAS, HRS, and SHARE were investigated to explore potential effect modifications.

Subsequently, patients with prior CMDs or CMM in the CHARLS, MHAS, HRS, and SHARE were included to explore the associations between internet use and death. The mediating role of depressive symptoms was also explored.

Random-effects meta-analysis was used to pool the subdistribution hazard ratios (sHRs) and corresponding 95% confidence intervals (CIs) from different cohorts to derive overall effect estimates. The heterogeneity of effect estimated across cohorts was tested using the Cochran Q test and I^2^ statistic.

Reporting of this study was done under Strengthening the Reporting of Observational studies in Epidemiology (STROBE) guidelines. Analyses were performed using SAS statistical software version 9.4 (SAS Institute) and R statistical software version 4.2.3 (R Project for Statistical Computing). All analyses were two-sided, and the *P* values of < 0.05 and 95% CIs that did not cross 1.00 were considered statistically significant.

## Results

The baseline characteristics of the included participants in the CHARLS, MHAS, HRS, and SHARE are shown in Table 1.

**Table 1:** Characteristics of included participants in the CHARLS, MHAS, HRS, and SHARE

Characteristics		CHARLS		MHAS		HRS		SHARE	
		Digital exclusion (*N* = 12,102)	Internet use (*N* = 236)	Digital exclusion (*N* = 1,0239)	Internet use (*N* = 4,062)	Digital exclusion (*N* = 8,269)	Internet use (*N* = 8,522)	Digital exclusion (*N* = 29,862)	Internet use (*N* = 31,130)
Age, years		61.0 (56.0–68.0)	57.0 (53.0-61.5)	65.0 (59.0–73.0)	62.0 (56.0–68.0)	71.0 (60.0–79.0)	62.0 (56.0–70.0)	72.0 (64.0–79.0)	62.0 (56.0–68.0)
Sex									
	Men	5910 (48.8)	143 (60.6)	4443 (43.4)	1833 (45.1)	3410 (41.2)	3598 (42.2)	12,034 (40.3)	15,104 (48.5)
	Women	6192 (51.2)	93 (39.4)	5796 (56.6)	2229 (54.9)	4859 (58.8)	4924 (57.8)	17,828 (59.7)	16,026 (51.5)
Education									
	Less than high school	10,886 (89.9)	76 (32.2)	9673 (94.5)	2658 (65.4)	3370 (40.8)	744 (8.7)	17,500 (58.6)	6446 (20.7)
	High school	739 (6.1)	52 (22.0)	188 (1.8)	284 (7.0)	2787 (33.7)	2027 (23.8)	8323 (27.9)	11,272 (36.2)
	College or above	469 (3.9)	108 (45.8)	336 (3.3)	1100 (27.1)	2109 (25.5)	5751 (67.5)	3615 (12.1)	13,044 (41.9)
	Missing	8 (0.1)	0 (0.0)	42 (0.4)	20 (0.5)	3 (0.0)	0 (0.0)	424 (1.4)	368 (1.2)
Household income									
	First tertile	3507 (29.0)	10 (4.2)	4114 (40.2)	956 (23.5)	4245 (51.3)	1348 (15.8)	11,509 (38.5)	4692 (15.1)
	Second tertile	3492 (28.8)	23 (9.8)	3449 (33.7)	746 (18.4)	2846 (34.4)	2745 (32.2)	7898 (26.5)	8323 (26.7)
	Third tertile	3359 (27.8)	165 (69.9)	2418 (23.6)	2213 (54.5)	1178 (14.3)	4429 (52.0)	4120 (13.8)	12,046 (38.7)
	Missing	1744 (14.4)	38 (16.1)	258 (2.5)	147 (3.6)			6335 (21.2)	6069 (19.5)
Marital status									
	Married/cohabiting	10,267 (84.8)	219 (92.8)	5970 (58.3)	2892 (71.2)	4406 (53.3)	6202 (72.8)	19,697 (66.0)	24,805 (79.7)
	Single	1834 (15.2)	17 (7.2)	4269 (41.7)	1170 (28.8)	3851 (46.5)	2317 (27.2)	10,165 (34.0)	6325 (20.3)
	Missing	1 (0.0)	0 (0.0)			12 (0.2)	3 (0.0)		
Current smoking									
	No	8316 (68.7)	165 (69.9)	9058 (88.5)	3525 (86.8)	6764 (81.8)	7435 (87.2)	24,913 (83.4)	25,286 (81.2)
	Yes	3781 (31.2)	71 (30.1)	1178 (11.5)	535 (13.2)	1450 (17.5)	1047 (12.3)	4941 (16.6)	5842 (18.8)
	Missing	5 (0.1)	0 (0.0)	3 (0.0)	2 (0.1)	55 (0.7)	40 (0.5)	8 (0.0)	2 (0.0)
Current drinking									
	No	9090 (75.1)	159 (67.4)	8796 (85.9)	3190 (78.5)	6071 (73.4)	4491 (52.7)	16,468 (55.1)	8894 (28.5)
	Yes	3008 (24.9)	77 (32.6)	1398 (13.7)	849 (20.9)	2170 (26.3)	4015 (47.1)	13,377 (44.8)	22,220 (71.4)
	Missing	4 (0.0)	0 (0.0)	45 (0.4)	23 (0.6)	28 (0.3)	16 (0.2)	17 (0.1)	16 (0.1)
Abnormal weight									
	No	7233 (59.8)	90 (38.1)	3120 (30.5)	1063 (26.2)	2514 (30.4)	2229 (26.1)	10,087 (33.8)	12,478 (40.1)
	Yes	2954 (24.4)	53 (22.5)	5583 (54.5)	2806 (69.1)	5585 (67.5)	6211 (72.9)	18,618 (62.4)	18,224 (58.5)
	Missing	1915 (15.8)	93 (39.4)	1536 (15.0)	193 (4.8)	170 (2.1)	82 (1.0)	1157 (3.9)	428 (1.4)
Hypertension									
	No	5484 (45.3)	128 (54.2)	4768 (46.6)	2149 (52.9)	2727 (33.0)	4120 (48.3)	16,034 (53.7)	20,904 (67.2)
	Yes	6618 (54.7)	108 (45.8)	5471 (53.4)	1913 (47.1)	5542 (67.0)	4402 (51.7)	13,828 (46.3)	10,226 (32.8)
Depressive symptoms*									
	No	6909 (60.9)	204 (88.7)	5952 (63.8)	2990 (79.0)	5988 (78.9)	7443 (89.4)	19,034 (66.8)	25,045 (81.6)
	Yes	4435 (39.1)	26 (11.3)	3384 (36.3)	797 (21.1)	1601 (21.1)	884 (10.6)	9451 (33.2)	5639 (18.4)
New-onset CMM*									
	No	10,767 (98.0)	896 (98.9)	9224 (71.6)	644 (70.0)	6268 (46.1)	719 (56.4)	26,596 (47.2)	1487 (68.1)
	Yes	216 (2.0)	10 (1.1)	3661 (28.4)	276 (30.0)	7340 (53.9)	556 (43.6)	29,741 (52.8)	698 (32.0)

Notes: CHARLS, China Health and Retirement Longitudinal Study. MHAS, Mexican Health and Aging Study. HRS, Health and Retirement Study. SHARE, Survey of Health, Ageing and Retirement in Europe. CMM, cardiometabolic multimorbidity. *Indicates that the analytic samples were less than the included participants

A total of 12,338 participants from the CHARLS, 14,301 participants from the MHAS, 16,791 participants from the HRS, and 60,992 participants from the SHARE were included. In all four cohorts, younger adults and those with higher socioeconomic status (higher education and household income) tend to exhibit higher internet usage.

According to Table 2, internet use (vs. digital exclusion) is associated with a lower risk of diabetes, stroke, and death, with pooled sHRs (95% CIs) of 0.83 (0.74–0.93), 0.81 (0.71–0.92), and 0.67 (0.52–0.86), respectively. In the two developing countries, older internet users (vs. digital exclusion) in China are 50% less likely to develop new-onset CMM, but those in Mexico are not at any lower risk of CMDs or CMM. For the developed countries, older internet users (vs. digital exclusion) in the United States are less likely to develop new-onset diabetes and death, with sHR (95% CI) of 0.87 (0.77–0.99) and 0.58 (0.52–0.65); interestingly, those in Europe are at 14-30% lower risk of all CMDs and CMM and 40% lower risk of death. The findings of sensitivity analysis in Table S1 are similar to our main findings. Furthermore, depressive symptoms may play a mediating role in the association of internet use with new-onset CMDs, CMM, and death, especially in developed countries. For instance, depressive symptoms may mediate 6.3-14.5% of the association of internet use with new-onset CMDs and CMM, as well as 5.8% of that between internet use and death (Table 2).

**Table 2:** Association of internet use with new-onset CMDs, CMM, and all-cause death and the mediating role of depressive symptoms

	CHARLS	MHAS	HRS	SHARE	Pooled	*P* value for heterogeneity
**Diabetes**						
sHR (95% CI)	0.70 (0.44–1.12)	0.92 (0.81–1.05)	0.87 (0.77–0.99)	0.76 (0.70–0.83)	0.83 (0.74–0.93)	0.061
Mediation, % (95% CI)	11.7 (4.3–62.5)		4.1 (2.1–7.6)	6.3 (4.4–8.9)		
*P* values	0.032	0.520	< 0.001	< 0.001		
**Heart diseases**						
sHR (95% CI)	0.92 (0.62–1.34)	1.05 (0.93–1.19)	1.07 (0.95–1.21)	0.86 (0.80–0.92)	0.97 (0.86–1.11)	0.003
Mediation, % (95% CI)				14.5 (10.3–22.7)		
*P* values	0.062	0.108	0.098	< 0.001		
**Stroke**						
sHR (95% CI)	0.70 (0.38–1.28)	0.99 (0.75–1.30)	0.84 (0.70–1.01)	0.75 (0.67–0.84)	0.81 (0.71–0.92)	0.264
Mediation, % (95% CI)			5.6 (3.0-11.3)	8.6 (6.2–12.3)		
*P* values	0.778	0.338	< 0.001	< 0.001		
**CMM**						
sHR (95% CI)	0.50 (0.27–0.94)	1.12 (0.96–1.30)	0.90 (0.79–1.04)	0.70 (0.62–0.78)	0.83 (0.63–1.09)	< 0.001
Mediation, % (95% CI)	17.8 (6.8–99.6)		12.0 (5.6–39.2)	10.2 (7.2–14.1)		
*P* values	0.036	0.076	0.004	< 0.001		
**All-cause death**						
sHR (95% CI)	0.56 (0.27–1.15)	0.91 (0.81–1.02)	0.58 (0.52–0.65)	0.60 (0.56–0.64)	0.67 (0.52–0.86)	< 0.001
Mediation, % (95% CI)		12.2 (5.5–46.2)	4.0 (2.3–6.7)	5.8 (4.6–7.2)		
*P* values	0.230	0.010	< 0.001	< 0.001		

Notes: CMD, cardiometabolic diseases. CMM, cardiometabolic multimorbidity. CHARLS, China Health and Retirement Longitudinal Study. MHAS, Mexican Health and Aging Study. HRS, Health and Retirement Study. SHARE, Survey of Health, Ageing and Retirement in Europe. sHR, subdistribution hazard ratio. CI, confidence interval. All models were adjusted for age, sex, education, household income, marital status, current smoking, current drinking, abnormal weight, and hypertension

**Fig. 2:**
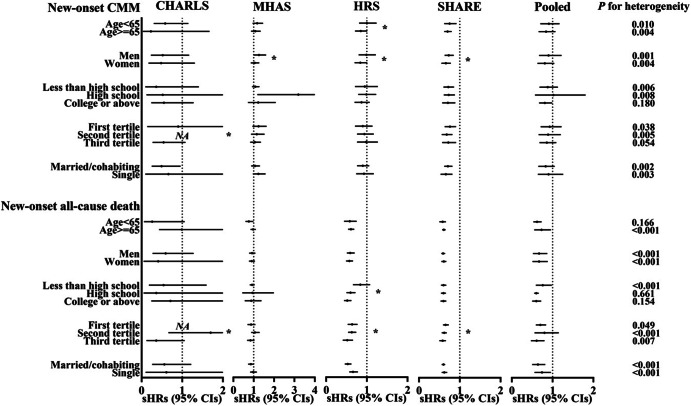
Stratified association of internet use with new-onset CMM and all-cause death. Notes: CMM, cardiometabolic multimorbidity. CHARLS, China Health and Retirement Longitudinal Study. MHAS, Mexican Health and Aging Study. HRS, Health and Retirement Study. SHARE, Survey of Health, Ageing and Retirement in Europe. sHR, subdistribution hazard ratio. CI, confidence interval. All models were adjusted for age, sex, education, household income, marital status, current smoking, current drinking, abnormal weight, and hypertension. * referred to *P* for interaction < 0.05

Figure 2 and Table S2 indicate that the negative associations of internet use with new-onset CMM and death are more pronounced in individuals with the highest SES and women. However, in MHAS, internet use is associated with a higher risk of new-onset CMM among those with high school education (sHR = 3.19, 95% CI: 1.18–8.61) and is marginally significantly associated with new-onset CMM among men (sHR = 1.26, 95% CI: 0.99–1.61, *P* for interaction = 0.025). Certain positive associations of internet use with new-onset CMDs were also found in stratified analyses. For instance, younger internet users in HRS are more likely to develop heart diseases (sHR = 1.23, 95% CI: 1.02–1.48, *P* for interaction = 0.005) as shown in Table S3. Men, individuals with high school education, and those with the lowest income tertile in MHAS are worthy of attention (Table S4-S6). Furthermore, Table S7 indicates that single internet users in HRS are at a marginally significant risk of developing new-onset heart diseases, with a sHR (95% CI) of 1.20 (0.98–1.46).

According to Table 3, internet use is associated with a lower risk of death in patients with prior CMDs and CMM, with pooled sHRs (95% CIs) of 0.69 (0.53–0.90) and 0.70 (0.50–0.96), respectively. Notably, this protective association is more pronounced in developed countries (*P* value for heterogeneity < 0.001). Similarly, depressive symptoms may mediate the association between internet use and death in patients with CMDs or CMM in the HRS and SHARE, with mediation proportions and its’ 95% CIs of 4.8 (2.1–8.9) for CMDs and 6.1 (2.7–11.5) for CMM in the former and 8.2 (5.6–12.2) and 9.0 (2.6–25.5) for the SHARE.

**Table 3:** Association of internet use with all-cause death among patients with prior CMDs and the mediating role of depressive symptoms

		CHARLS	MHAS	HRS	SHARE	Pooled	*P* value for heterogeneity
**Prior diabetes**							
sHR (95% CI)			0.92 (0.77–1.10)	0.58 (0.48–0.70)	0.65 (0.57–0.74)	0.70 (0.54–0.92)	0.001
Mediation (% [95% CI])					7.3 (3.9–12.7)		
*P* values			0.182	0.062	< 0.001		
**Prior heart diseases**							
sHR (95% CI)		0.33 (0.05–2.38)	0.96 (0.71–1.31)	0.58 (0.49–0.68)	0.69 (0.62–0.78)	0.70 (0.54–0.90)	0.029
Mediation (% [95% CI])				4.6 (1.4–9.5)	9.8 (5.5–18.4)		
*P* values		0.872	0.660	0.008	< 0.001		
**Prior stroke**							
sHR (95% CI)			1.15 (0.82–1.62)	0.55 (0.43–0.72)	0.56 (0.45–0.69)	0.70 (0.44–1.11)	0.001
Mediation (% [95% CI])				5.8 (1.2–14.7)	7.9 (3.0–18.0)		
*P* values			0.934	0.014	< 0.001		
**Prior > = 1 CMD**							
sHR (95% CI)		0.21 (0.03–1.49)	0.93 (0.79–1.10)	0.59 (0.52–0.68)	0.65 (0.59–0.71)	0.69 (0.53–0.90)	< 0.001
Mediation (% [95% CI])				4.8 (2.1–8.9)	8.2 (5.6–12.2)		
*P* values		0.310	0.186	< 0.001	< 0.001		
**Prior CMM**							
sHR (95% CI)			1.00 (0.72–1.39)	0.55 (0.45–0.68)	0.66 (0.55–0.80)	0.70 (0.50–0.96)	0.010
Mediation (% [95% CI])				6.1 (2.7–11.5)	9.0 (2.6–25.5)		
*P* values			0.882	< 0.001	< 0.001		

Notes: CMD, cardiometabolic disease. CMM, cardiometabolic multimorbidity. CHARLS, China Health and Retirement Longitudinal Study. MHAS, Mexican Health and Aging Study. HRS, Health and Retirement Study. SHARE, Survey of Health, Ageing and Retirement in Europe. sHR, subdistribution hazard ratio. CI, confidence interval. All models were adjusted for age, sex, education, household income, marital status, current smoking, current drinking, abnormal weight, and hypertension

## Discussion

In this multi-cohort study and meta-analysis, we observed inverse associations between internet use and new-onset CMDs and CMM among elderly individuals. In addition, our findings suggest that internet use can help prevent death in both the general elderly population and patients with prior CMDs or CMM. The protective role of internet use is likely mediated by reduced depressive symptoms and is more pronounced in those with high SES and women. However, it is worth noting that there may be potential cardiometabolic risks attributed to internet use, particularly in older adults with low SES and men in developing countries.

Our study revealed that older adults who use the internet were at a lower risk of developing CMDs and death, which could be attributed to a decrease in depressive symptoms. Social support provided by internet use can be utilized to interpret this finding. Social isolation is a well-known risk factor for depressive symptoms, CMDs, and death among older adults [20, 21]. By facilitating new social connections and maintaining existing ones, internet use could reduce social isolation and depressive symptoms, thereby decreasing the risks of CMDs and death. Additionally, internet use among older adults could promote healthy lifestyles by facilitating access to health information and health education. Prior research has shown that older adults who use the internet are more likely to cultivate healthy lifestyles, which is a crucial component in preventing depressive symptoms, CMDs, and death [10, 12, 22]. Moreover, internet use could provide easier access to health information and encourage engagement in health decision-making, leading to older adults prioritizing their physical and mental well-being and seeking timely medical advices and treatments [5].

The inverse associations of internet use with new-onset CMDs, CMM, and death are more pronounced in developed countries. In comparison to developing countries, developed countries generally have more well-developed health service systems and stronger health promotion campaigns, which contribute to higher health literacy among older adults [23, 24]. Combined with their higher internet usage, older adults in developed countries are more likely to access health information and avoid health risks. Additionally, developed countries often have more comprehensive healthcare and public health infrastructures, which facilitate better access to preventive care, early disease detection, timely treatment, and advanced care. Healthcare providers in these areas may also be more likely to rely on internet-based resources and tools to support their patients’ health. On the other hand, the difference between developing and developed countries can be interpreted as differences in SES. For instance, we found that China and Mexico lagged far behind the United States and Europe in educational attainment. This study also demonstrates a more pronounced protective role of internet use in older adults with higher SES. Research has consistently shown that high SES is a protective factor against CMDs and death [25]. Older adults with high SES are more likely to have access to and make use of internet services for health information due to their better-educated backgrounds and financial advantages [23, 24]. Therefore, they are more likely to adopt healthier lifestyles, such as maintaining healthy diets, engaging in regular exercise, and abstaining from smoking and excessive alcohol consumption, all of which can contribute to a reduced risk of CMDs and death [12]. Furthermore, individuals with higher SES tend to prioritize their health and have better access to high-quality medical resources such as medical facilities and insurance, which can lead to early detection and timely treatment of CMDs, ultimately promoting better health outcomes [26].

Despite the inverse association between internet use and CMDs in general, we revealed certain increased cardiometabolic risks of internet use for individuals with lower SES and men in Mexico. This study positions Mexico as an intermediate state between China and developed countries owing to its lower education levels, yet relatively higher internet usage among the elderly. Our results suggest that internet use is more likely to be problematic, such as developing internet addiction, among older adults with lower SES or health literacy, which is supported by prior evidence [27]. Furthermore, internet users aged less than 65 years and single ones in the HRS also exhibit potential cardiometabolic risks. One common feature that distinguishes Mexican and American internet users from those in China and European countries is the higher rate of abnormal weight, according to our findings. Therefore, we call on internet users to focus on reducing sedentary behavior and strengthening exercise to control weight, so as to prevent cardiometabolic risks caused by overdose internet use, especially in those aged < 65 years and single.

Given the growing global attention around internet usage among older adults in recent years [28], our findings underscore the importance of effectively managing problematic internet use in older individuals with low SES or limited health literacy in developed countries. Additionally, policymakers in developing countries should take into account the delicate balance between internet usage and health literacy by developing better health education or health-related web information when promoting internet usage among the elderly. For older adults with low SES, appropriate internet education to address their limited access to health information and the prevention of internet addiction after learning internet use should be emphasized. Reduction and prevention of underlying depressive symptoms and the promotion of healthy lifestyles should also be emphasized to reduce the risk of CMDs and death attributed to overdose internet use.

To the best of our knowledge, this is the first comprehensive population-based multi-cohort and meta-analysis investigating the association of internet use with new-onset CMDs, CMM, and death among the general elderly, as well as the association between internet use and death in old patients with CMDs or CMM, across different countries with varying levels of development and health literacy. Our findings underscore the health benefits of enhancing internet usage among older adults and the imperative to balance SES and health literacy in this process, especially in developing countries. The large nationally representative sample size from diverse developing or developed countries largely guarantees the reliability and generalizability of our findings. The mediation analysis and multiple stratified analysis also enrich our conclusions. Moreover, sensitivity analysis was conducted in our study to confirm the robustness of our results.

Despite these strengths, our study remains limited due to several shortcomings. The low internet usage and limited analytical samples in the CHARLS may reduce our statistical power. In order to maximize statistical power, we had to use blood samples to further define diabetes in the CHARLS, which may increase the heterogeneity among cohorts. Internet use in the MHAS was defined as having household electronics with internet access, which was different from the other three cohorts and may also increase heterogeneity. Furthermore, the CMDs were defined using self-reported physician diagnoses or treatments, which may cause misclassification bias.

## Conclusion

In conclusion, our study found that older internet users were at lower risks of CMDs, CMM, and death and that patients with prior CMDs or CMM were less likely to die if they used the internet, especially in developed countries and in individuals with higher SES and women. Moreover, the balance of internet use, SES, and health literacy should be considered when enhancing internet usage among the elderly to prevent problematic internet use.

### Electronic supplementary material

Below is the link to the electronic supplementary material.

Supplementary Material 1

## Data Availability

The data that support the findings of this study are available from the websites of China Health and Retirement Longitudinal Study at http://charls.pku.edu.cn/en, the websites of Mexican Health and Aging Study at http://www.mhasweb.org/, the websites of The Health and Retirement Study at https://hrs.isr.umich.edu/, and the websites of Survey of Health, Ageing and Retirement in Europe at https://share-eric.eu/.

## Abbreviations

BMI: Body mass index
CESD: Center for Epidemiological Studies Depression Scale
CHARLS: China Health and Retirement Longitudinal Study
CIs: Confidence intervals
CMDs: Cardiometabolic diseases
CMM: Cardiometabolic multimorbidity
EURO-D: Europe-depression
HRS: Health and Retirement Study
INEGI: National Institute of Statistics and Geography
INSP: National Institute of Public Health
IQRs: Interquartile ranges
MHAS: Mexican Health and Aging Study
SES: Socioeconomic status
SHARE: Survey of Health, Ageing and Retirement in Europe
sHR: Subdistribution hazard ratio
STROBE: Strengthening the Reporting of Observational studies in Epidemiology
YLLs: Years of life lost
