# Supplementary material for: Internet use, cardiometabolic multimorbidity, and death in older adults: a multi-cohort study spanning developing and developed countries
Source: Global Health. 2023 Nov 6;19:81. doi: 10.1186/s12992-023-00984-z (PMC10626678; doi:10.1186/s12992-023-00984-z)
Supplement: Supplementary file 1 — Supplementary Material 1 [file 12992_2023_984_MOESM1_ESM.docx]

Supplement

eMethods

[eMethods 2](#_Toc147870611)

Table Legends

[Table S1. Sensitivity analysis on the association of internet use with new-onset CMDs, CMM, and all-cause death 3](#_Toc147870612)

[Table S2. Stratified association of internet use with new-onset CMM and all-cause death 4](#_Toc147870613)

[Table S3. Age stratified association of internet use with new-onset CMDs 6](#_Toc147870614)

[Table S4. Sex stratified association of internet use with new-onset CMDs 7](#_Toc147870615)

[Table S5. Education stratified association of internet use with new-onset CMDs 8](#_Toc147870616)

[Table S6. Household income stratified association of internet use with new-onset CMDs 9](#_Toc147870617)

[Table S7. Marital status stratified association of internet use with new-onset CMDs 10](#_Toc147870618)

# eMethods

In the CHARLS, depressive symptoms were evaluated using the 10-item Center for Epidemiological Studies Depression Scale (CESD-10). Participants were asked to indicate how they felt and behaved during the past week, with response options ranging from "rarely or none of the time" (one day), to "some or a little of the time" (1-2 days), to "sometimes or a significant amount of the time" (3-4 days), to "most or all of the time" (5-7 days). Responses were scored 0-3, with a total depression score ranging from 0 to 30. A cutoff score of 10 or higher was considered indicative of depressive symptoms^1^.

In the MHAS, depressive symptoms were assessed using the CESD-9. Participants were asked to report on the presence of nine symptoms experienced in the previous week, including feeling depressed, feeling that everything was an effort, restless sleep, feeling happy (reverse scored), feeling lonely, enjoying life (reverse scored), feeling sad, feeling tired, and having a lot of energy (reverse-scored), with a total score ranging from 0 to 9. A cutoff score of 5 or higher was considered to have depressive symptoms^2^.

In the HRS, depressive symptoms were measured by the CESD-8, which contains six negative indicators and two positive indicators. The negative indicators asked whether the respondent experienced the following six emotions all or most of the time during the last week: 1) depressed; 2) everything is an effort; 3) sleep is restless; 4) felt alone; 5) felt sad; and 6) could not get going. The positive indicators asked whether the respondent felt happy or enjoyed life all or most of the time during the past week. The response “yes” to each negative indicator and the response “no” to each positive indicator obtained one point. The total depressive score was calculated with a range of 0-8, with a cutoff score of 4 or higher indicating the presence of depressive symptoms^3^.

In the SHARE study, depressive symptoms were measured using the Europe-depression (EURO-D) scale, which surveyed 12 emotional states experienced by respondents within the previous month, including depressed mood, pessimism, suicidal tendencies, guilt, sleep, interest, irritability, appetite, fatigue, concentration, enjoyment, and tearfulness. Each item was scored as 0 or 1, with the total score ranging from 0 to 12. A score of 4 or higher was identified as depressive symptoms^4^.

**References**

1. Lei X, Sun X, Strauss J, Zhang P, Zhao Y. Depressive symptoms and SES among the mid-aged and elderly in China: evidence from the China Health and Retirement Longitudinal Study national baseline. *Soc Sci Med*. 2014;120:224-32. doi:10.1016/j.socscimed.2014.09.028

2. Aguilar-Navarro SG, Fuentes-Cantú A, Avila-Funes JA, García-Mayo EJ. Validity and reliability of the screening questionnaire for geriatric depression used in the Mexican Health and Age Study. *Salud Publica Mex*. 2007;49(4):256-62. Validez y confiabilidad del cuestionario del ENASEM para la depresión en adultos mayores. doi:10.1590/s0036-36342007000400005

3. Steffick DE, Wallace RB, Herzog ARJAA, MI: University of Michigan. Documentation of affective functioning measures in the Health and Retirement Study. 2000:15.

4. Prince MJ, Reischies F, Beekman ATF, et al. Development of the EURO–D scale – a European Union initiative to compare symptoms of depression in 14 European centres. *British Journal of Psychiatry*. 1999;174(4):330-338. doi:10.1192/bjp.174.4.330

# Table S1. Sensitivity analysis on the association of internet use with new-onset CMDs, CMM, and all-cause death

|  | **CHARLS** | **MHAS** | **HRS** | **SHARE** | **Pooled** | ***P* value for heterogeneity** |
| --- | --- | --- | --- | --- | --- | --- |
|  | **sHR (95% CI)** | | | | |  |
|  | **Sensitivity analysis 1: complete-case analysis** | | | | | |
| Diabetes | 0.66 (0.35-1.25) | 0.90 (0.79-1.03) | 0.87 (0.76-0.99) | 0.75 (0.68-0.83) | 0.83 (0.74-0.92) | 0.103 |
| Heart diseases | 0.99 (0.62-1.59) | 1.03 (0.90-1.17) | 1.07 (0.95-1.21) | 0.85 (0.79-0.92) | 0.97 (0.85-1.11) | 0.005 |
| Stroke | 0.62 (0.26-1.50) | 0.96 (0.71-1.29) | 0.84 (0.70-1.01) | 0.79 (0.70-0.89) | 0.82 (0.74-0.90) | 0.595 |
| CMM | 0.53 (0.23-1.19) | 1.09 (0.93-1.28) | 0.90 (0.78-1.04) | 0.71 (0.63-0.80) | 0.85 (0.67-1.08) | <0.001 |
| All-cause death | 0.40 (0.14-1.18) | 0.96 (0.85-1.09) | 0.58 (0.52-0.65) | 0.60 (0.56-0.64) | 0.67 (0.49-0.90) | <0.001 |
|  | **Sensitivity analysis 2: excluding new-onset conditions within the next wave** | | | | | |
| Diabetes | 0.80 (0.49-1.29) | 0.92 (0.81-1.05) | 0.85 (0.73-0.98) | 0.80 (0.71-0.91) | 0.85 (0.79-0.93) | 0.494 |
| Heart diseases | 0.83 (0.53-1.30) | 1.06 (0.94-1.20) | 1.07 (0.93-1.23) | 0.87 (0.80-0.96) | 0.98 (0.86-1.11) | 0.021 |
| Stroke | 0.80 (0.44-1.46) | 0.99 (0.76-1.30) | 0.79 (0.64-0.97) | 0.74 (0.64-0.85) | 0.80 (0.70-0.92) | 0.317 |
| CMM | 0.61 (0.32-1.15) | 1.12 (0.97-1.31) | 0.87 (0.74-1.03) | 0.74 (0.64-0.86) | 0.87 (0.69-1.08) | 0.001 |
| All-cause death | 0.68 (0.33-1.41) | 0.87 (0.76-0.99) | 0.66 (0.57-0.76) | 0.63 (0.58-0.67) | 0.71 (0.59-0.85) | <0.001 |

Notes: CMD, cardiometabolic diseases. CMM, cardiometabolic multimorbidity. CHARLS, China Health and Retirement Longitudinal Study. MHAS, Mexican Health and Aging Study. HRS, Health and Retirement Study. SHARE, Survey of Health, Ageing and Retirement in Europe. sHR, subdistribution hazard ratio. CI, confidence interval. All models were adjusted for age, sex, education, household income, marital status, current smoking, current drinking, abnormal weight, and hypertension.

# Table S2. Stratified association of internet use with new-onset CMM and all-cause death

|  | | **CHARLS** | **MHAS** | **HRS** | **SHARE** | **Pooled** | ***P* value for heterogeneity** |
| --- | --- | --- | --- | --- | --- | --- | --- |
|  |  | **sHR (95% CI)** | | | | |  |
|  |  | **New-onset CMM** | | | | |  |
| **Age-stratified** | |  |  |  |  |  |  |
|  | *P* for interaction | 0.474 | 0.920 | 0.022 | 0.056 |  |  |
|  | Age<65 | 0.58 (0.30-1.15) | 1.17 (0.95-1.46) | 1.00 (0.81-1.24) | 0.75 (0.62-0.92) | 0.91 (0.70-1.17) | 0.010 |
|  | Age>=65 | 0.23 (0.03-1.67) | 1.08 (0.87-1.34) | 0.85 (0.71-1.01) | 0.70 (0.62-0.80) | 0.84 (0.66-1.07) | 0.004 |
| **Sex-stratified** | |  |  |  |  |  |  |
|  | *P* for interaction | 0.754 | 0.025 | 0.015 | 0.007 |  |  |
|  | Men | 0.52 (0.23-1.17) | 1.26 (0.99-1.61) | 0.99 (0.80-1.22) | 0.73 (0.63-0.85) | 0.90 (0.67-1.22) | 0.001 |
|  | Women | 0.48 (0.17-1.31) | 1.05 (0.86-1.27) | 0.84 (0.70-1.01) | 0.66 (0.55-0.78) | 0.81 (0.63-1.04) | 0.004 |
| **Education** | |  |  |  |  |  |  |
|  | *P* for interaction | 0.793 | 0.201 | 0.128 | 0.264 |  |  |
|  | Less than high school | 0.36 (0.09-1.41) | 1.08 (0.92-1.28) | 0.95 (0.71-1.27) | 0.72 (0.60-0.86) | 0.88 (0.68-1.13) | 0.006 |
|  | High school | 0.52 (0.12-2.20) | 3.19 (1.18-8.61) | 0.99 (0.79-1.23) | 0.73 (0.61-0.88) | 1.02 (0.57-1.81) | 0.008 |
|  | College or above | 0.55 (0.24-1.28) | 1.22 (0.72-2.08) | 0.87 (0.70-1.07) | 0.71 (0.57-0.89) | 0.81 (0.67-0.99) | 0.180 |
| **Household income** | |  |  |  |  |  |  |
|  | *P* for interaction | <0.001 | 0.437 | 0.375 | 0.189 |  |  |
|  | First tertile | 0.90 (0.14-5.81) | 1.24 (0.94-1.63) | 0.90 (0.71-1.14) | 0.76 (0.63-0.91) | 0.93 (0.71-1.22) | 0.038 |
|  | Second tertile | - | 1.15 (0.87-1.53) | 0.94 (0.76-1.17) | 0.68 (0.56-0.82) | 0.89 (0.66-1.20) | 0.005 |
|  | Third tertile | 0.54 (0.27-1.08) | 1.06 (0.83-1.35) | 0.98 (0.76-1.27) | 0.72 (0.57-0.91) | 0.86 (0.68-1.09) | 0.054 |
| **Marital status** | |  |  |  |  |  |  |
|  | *P* for interaction | 0.775 | 0.246 | 0.383 | 0.843 |  |  |
|  | Married/cohabiting | 0.49 (0.25-0.96) | 1.07 (0.88-1.30) | 0.90 (0.76-1.06) | 0.71 (0.62-0.82) | 0.83 (0.65-1.05) | 0.002 |
|  | Single | 0.66 (0.09-4.78) | 1.23 (0.96-1.58) | 0.93 (0.74-1.17) | 0.66 (0.53-0.82) | 0.90 (0.64-1.26) | 0.003 |
|  |  | **New-onset all-cause death** | | | | | |
| **Age-stratified** | |  |  |  |  |  |  |
|  | *P* for interaction | 0.097 | 0.365 | 0.345 | 0.357 |  |  |
|  | Age<65 | 0.26 (0.06-1.06) | 0.76 (0.60-0.98) | 0.58 (0.44-0.75) | 0.58 (0.50-0.66) | 0.62 (0.53-0.73) | 0.166 |
|  | Age>=65 | 0.99 (0.43-2.29) | 0.97 (0.85-1.10) | 0.61 (0.54-0.69) | 0.61 (0.57-0.65) | 0.73 (0.55-0.96) | <0.001 |
| **Sex-stratified** | |  |  |  |  |  |  |
|  | *P* for interaction | 0.536 | 0.348 | 0.382 | 0.089 |  |  |
|  | Men | 0.59 (0.27-1.28) | 0.92 (0.78-1.08) | 0.59 (0.51-0.70) | 0.59 (0.55-0.64) | 0.67 (0.52-0.87) | <0.001 |
|  | Women | 0.41 (0.05-3.14) | 0.90 (0.77-1.06) | 0.57 (0.49-0.66) | 0.61 (0.55-0.67) | 0.67 (0.51-0.88) | <0.001 |
| **Education** | |  |  |  |  |  |  |
|  | *P* for interaction | 0.889 | 0.654 | 0.008 | 0.254 |  |  |
|  | Less than high school | 0.54 (0.18-1.60) | 0.92 (0.81-1.04) | 0.84 (0.66-1.08) | 0.60 (0.54-0.67) | 0.76 (0.59-0.98) | <0.001 |
|  | High school | 0.36 (0.05-2.83) | 0.94 (0.44-1.99) | 0.60 (0.50-0.72) | 0.60 (0.54-0.66) | 0.60 (0.55-0.66) | 0.661 |
|  | College or above | 0.71 (0.24-2.13) | 0.88 (0.56-1.38) | 0.52 (0.44-0.62) | 0.60 (0.53-0.67) | 0.60 (0.50-0.72) | 0.154 |
| **Household income** | |  |  |  |  |  |  |
|  | *P* for interaction | <0.001 | 0.082 | 0.029 | <0.001 |  |  |
|  | First tertile | - | 0.87 (0.70-1.08) | 0.64 (0.53-0.77) | 0.65 (0.59-0.73) | 0.70 (0.59-0.84) | 0.049 |
|  | Second tertile | 1.70 (0.66-4.39) | 1.06 (0.88-1.29) | 0.63 (0.54-0.74) | 0.62 (0.56-0.68) | 0.80 (0.56-1.15) | <0.001 |
|  | Third tertile | 0.36 (0.12-1.06) | 0.84 (0.68-1.03) | 0.52 (0.41-0.66) | 0.58 (0.50-0.66) | 0.61 (0.47-0.80) | 0.007 |
| **Marital status** | |  |  |  |  |  |  |
|  | *P* for interaction | 0.918 | 0.765 | 0.094 | 0.443 |  |  |
|  | Married/cohabiting | 0.56 (0.26-1.22) | 0.86 (0.73-1.01) | 0.53 (0.46-0.62) | 0.60 (0.56-0.64) | 0.64 (0.50-0.82) | <0.001 |
|  | Single | 0.61 (0.10-3.84) | 0.97 (0.83-1.15) | 0.66 (0.57-0.78) | 0.62 (0.56-0.69) | 0.74 (0.56-0.96) | <0.001 |

Notes: CMM, cardiometabolic multimorbidity. CHARLS, China Health and Retirement Longitudinal Study. MHAS, Mexican Health and Aging Study. HRS, Health and Retirement Study. SHARE, Survey of Health, Ageing and Retirement in Europe. sHR, subdistribution hazard ratio. CI, confidence interval. All models were adjusted for age, sex, education, household income, marital status, current smoking, current drinking, abnormal weight, and hypertension.

# Table S3. Age stratified association of internet use with new-onset CMDs

|  | | **CHARLS** | **MHAS** | **HRS** | **SHARE** | **Pooled** | ***P* value for heterogeneity** |
| --- | --- | --- | --- | --- | --- | --- | --- |
|  |  | **sHR (95% CI)** | | | | |  |
| **Diabetes** | |  |  |  |  |  |  |
|  | *P* for interaction | 0.126 | 0.298 | 0.105 | 0.792 |  |  |
|  | Age<65 | 0.81 (0.49-1.34) | 0.90 (0.77-1.06) | 0.84 (0.72-0.98) | 0.78 (0.69-0.89) | 0.83 (0.76-0.90) | 0.587 |
|  | Age>=65 | 0.36 (0.09-1.43) | 0.97 (0.77-1.22) | 0.91 (0.73-1.12) | 0.76 (0.68-0.85) | 0.84 (0.72-0.98) | 0.109 |
| **Heart diseases** | |  |  |  |  |  |  |
|  | *P* for interaction | 0.145 | 0.582 | 0.005 | 0.152 |  |  |
|  | Age<65 | 1.00 (0.65-1.54) | 1.09 (0.91-1.31) | 1.23 (1.02-1.48) | 0.89 (0.78-1.00) | 1.04 (0.88-1.23) | 0.030 |
|  | Age>=65 | 0.89 (0.38-2.07) | 1.04 (0.88-1.24) | 0.98 (0.83-1.14) | 0.86 (0.79-0.93) | 0.94 (0.83-1.05) | 0.172 |
| **Stroke** | |  |  |  |  |  |  |
|  | *P* for interaction | 0.688 | 0.376 | 0.282 | 0.131 |  |  |
|  | Age<65 | 0.83 (0.44-1.58) | 0.95 (0.64-1.42) | 1.02 (0.76-1.36) | 0.73 (0.60-0.89) | 0.86 (0.70-1.05) | 0.262 |
|  | Age>=65 | 0.30 (0.04-2.10) | 1.01 (0.69-1.47) | 0.80 (0.64-1.00) | 0.78 (0.68-0.89) | 0.80 (0.72-0.89) | 0.469 |

Notes: CMDs, cardiometabolic diseases. CMM, cardiometabolic multimorbidity. CHARLS, China Health and Retirement Longitudinal Study. MHAS, Mexican Health and Aging Study. HRS, Health and Retirement Study. SHARE, Survey of Health, Ageing and Retirement in Europe. sHR, subdistribution hazard ratio. CI, confidence interval. All models were adjusted for age, sex, education, household income, marital status, current smoking, current drinking, abnormal weight, and hypertension.

# Table S4. Sex stratified association of internet use with new-onset CMDs

|  | | **CHARLS** | **MHAS** | **HRS** | **SHARE** | **Pooled** | ***P* value for heterogeneity** |
| --- | --- | --- | --- | --- | --- | --- | --- |
|  |  | **sHR (95% CI)** | | | | |  |
| **Diabetes** | |  |  |  |  |  |  |
|  | *P* for interaction | 0.183 | <0.001 | 0.632 | <0.001 |  |  |
|  | Men | 0.75 (0.42-1.35) | 1.20 (0.99-1.47) | 0.80 (0.66-0.98) | 0.79 (0.70-0.90) | 0.89 (0.71-1.12) | 0.004 |
|  | Women | 0.62 (0.28-1.39) | 0.76 (0.64-0.91) | 0.92 (0.78-1.09) | 0.73 (0.64-0.83) | 0.79 (0.69-0.90) | 0.163 |
| **Heart diseases** | |  |  |  |  |  |  |
|  | *P* for interaction | 0.097 | 0.212 | 0.457 | <0.001 |  |  |
|  | Men | 0.81 (0.47-1.38) | 1.07 (0.87-1.31) | 1.05 (0.87-1.27) | 0.94 (0.85-1.03) | 0.98 (0.90-1.06) | 0.495 |
|  | Women | 1.07 (0.62-1.84) | 1.05 (0.89-1.23) | 1.08 (0.92-1.27) | 0.77 (0.69-0.86) | 0.96 (0.79-1.16) | 0.001 |
| **Stroke** | |  |  |  |  |  |  |
|  | *P* for interaction | 0.413 | 0.678 | 0.866 | 0.066 |  |  |
|  | Men | 0.50 (0.21-1.18) | 1.00 (0.66-1.51) | 0.84 (0.63-1.11) | 0.82 (0.70-0.95) | 0.83 (0.73-0.94) | 0.545 |
|  | Women | 1.09 (0.46-2.54) | 1.00 (0.69-1.44) | 0.86 (0.68-1.09) | 0.68 (0.57-0.81) | 0.82 (0.66-1.01) | 0.149 |

Notes: CMDs, cardiometabolic diseases. CMM, cardiometabolic multimorbidity. CHARLS, China Health and Retirement Longitudinal Study. MHAS, Mexican Health and Aging Study. HRS, Health and Retirement Study. SHARE, Survey of Health, Ageing and Retirement in Europe. sHR, subdistribution hazard ratio. CI, confidence interval. All models were adjusted for age, education, household income, marital status, current smoking, current drinking, abnormal weight, and hypertension.

# Table S5. Education stratified association of internet use with new-onset CMDs

|  | | **CHARLS** | **MHAS** | **HRS** | **SHARE** | **Pooled** | ***P* value for heterogeneity** |
| --- | --- | --- | --- | --- | --- | --- | --- |
|  |  | **sHR (95% CI)** | | | | |  |
| **Diabetes** | |  |  |  |  |  |  |
|  | *P* for interaction | 0.388 | 0.429 | 0.626 | 0.292 |  |  |
|  | Less than high school | 0.56 (0.24-1.33) | 0.95 (0.83-1.09) | 0.83 (0.65-1.08) | 0.70 (0.61-0.81) | 0.81 (0.67-0.97) | 0.018 |
|  | High school | 0.45 (0.11-1.90) | 1.05 (0.56-1.95) | 1.02 (0.82-1.27) | 0.83 (0.72-0.96) | 0.90 (0.76-1.07) | 0.316 |
|  | College or above | 0.94 (0.50-1.78) | 0.73 (0.50-1.07) | 0.81 (0.67-0.98) | 0.79 (0.65-0.96) | 0.80 (0.70-0.90) | 0.919 |
| **Heart diseases** | |  |  |  |  |  |  |
|  | *P* for interaction | 0.223 | 0.169 | 0.019 | 0.443 |  |  |
|  | Less than high school | 1.09 (0.56-2.10) | 1.02 (0.89-1.17) | 1.17 (0.91-1.52) | 0.80 (0.71-0.90) | 0.97 (0.80-1.18) | 0.011 |
|  | High school | 1.25 (0.62-2.52) | 2.24 (1.05-4.75) | 1.09 (0.89-1.33) | 0.92 (0.82-1.04) | 1.08 (0.86-1.37) | 0.065 |
|  | College or above | 0.64 (0.34-1.17) | 1.07 (0.71-1.63) | 1.06 (0.89-1.27) | 0.93 (0.81-1.07) | 0.97 (0.87-1.08) | 0.353 |
| **Stroke** | |  |  |  |  |  |  |
|  | *P* for interaction | 0.342 | 0.907 | 0.009 | 0.075 |  |  |
|  | Less than high school | 0.78 (0.29-2.08) | 1.03 (0.77-1.37) | 0.92 (0.61-1.36) | 0.76 (0.63-0.91) | 0.86 (0.71-1.05) | 0.350 |
|  | High school | - | 1.22 (0.19-7.89) | 1.05 (0.77-1.43) | 0.79 (0.66-0.94) | 0.89 (0.68-1.16) | 0.273 |
|  | College or above | 0.91 (0.42-2.00) | 0.87 (0.37-2.07) | 0.73 (0.56-0.94) | 0.76 (0.62-0.94) | 0.76 (0.65-0.89) | 0.942 |

Notes: CMDs, cardiometabolic diseases. CMM, cardiometabolic multimorbidity. CHARLS, China Health and Retirement Longitudinal Study. MHAS, Mexican Health and Aging Study. HRS, Health and Retirement Study. SHARE, Survey of Health, Ageing and Retirement in Europe. sHR, subdistribution hazard ratio. CI, confidence interval. All models were adjusted for age, sex, household income, marital status, current smoking, current drinking, abnormal weight, and hypertension.

# Table S6. Household income stratified association of internet use with new-onset CMDs

|  | | **CHARLS** | **MHAS** | **HRS** | **SHARE** | **Pooled** | ***P* value for heterogeneity** |
| --- | --- | --- | --- | --- | --- | --- | --- |
|  |  | **sHR (95% CI)** | | | | |  |
| **Diabetes** | |  |  |  |  |  |  |
|  | *P* for interaction | 0.544 | 0.195 | 0.478 | 0.001 |  |  |
|  | First tertile | - | 1.22 (0.98-1.53) | 0.92 (0.74-1.13) | 0.89 (0.77-1.04) | 0.99 (0.82-1.19) | 0.062 |
|  | Second tertile | 0.26 (0.03-1.98) | 0.88 (0.67-1.15) | 0.85 (0.70-1.03) | 0.71 (0.60-0.84) | 0.79 (0.69-0.91) | 0.287 |
|  | Third tertile | 0.84 (0.51-1.38) | 0.78 (0.65-0.95) | 0.91 (0.71-1.16) | 0.70 (0.58-0.84) | 0.78 (0.69-0.88) | 0.406 |
| **Heart diseases** | |  |  |  |  |  |  |
|  | *P* for interaction | 0.880 | 0.619 | 0.171 | 0.161 |  |  |
|  | First tertile | 0.59 (0.09-3.85) | 1.13 (0.90-1.42) | 1.11 (0.90-1.35) | 0.84 (0.74-0.94) | 0.99 (0.81-1.21) | 0.033 |
|  | Second tertile | 0.78 (0.23-2.61) | 1.08 (0.86-1.37) | 1.16 (0.96-1.40) | 0.88 (0.78-0.98) | 1.01 (0.85-1.20) | 0.066 |
|  | Third tertile | 0.95 (0.62-1.46) | 1.01 (0.83-1.22) | 1.02 (0.82-1.26) | 0.92 (0.79-1.06) | 0.97 (0.87-1.07) | 0.830 |
| **Stroke** | |  |  |  |  |  |  |
|  | *P* for interaction | 0.827 | 0.973 | 0.239 | 0.632 |  |  |
|  | First tertile | 0.87 (0.11-6.76) | 1.12 (0.71-1.75) | 0.72 (0.52-1.00) | 0.66 (0.54-0.82) | 0.77 (0.59-1.00) | 0.223 |
|  | Second tertile | - | 0.97 (0.57-1.64) | 1.03 (0.78-1.37) | 0.73 (0.60-0.89) | 0.87 (0.67-1.12) | 0.121 |
|  | Third tertile | 0.76 (0.40-1.46) | 0.93 (0.58-1.48) | 0.84 (0.60-1.18) | 0.98 (0.75-1.29) | 0.91 (0.76-1.09) | 0.848 |

Notes: CMDs, cardiometabolic diseases. CMM, cardiometabolic multimorbidity. CHARLS, China Health and Retirement Longitudinal Study. MHAS, Mexican Health and Aging Study. HRS, Health and Retirement Study. SHARE, Survey of Health, Ageing and Retirement in Europe. sHR, subdistribution hazard ratio. CI, confidence interval. All models were adjusted for age, sex, education, marital status, current smoking, current drinking, abnormal weight, and hypertension.

# Table S7. Marital status stratified association of internet use with new-onset CMDs

|  | | **CHARLS** | **MHAS** | **HRS** | **SHARE** | **Pooled** | ***P* value for heterogeneity** |
| --- | --- | --- | --- | --- | --- | --- | --- |
|  |  | **sHR (95% CI)** | | | | |  |
| **Diabetes** | |  |  |  |  |  |  |
|  | *P* for interaction | 0.199 | 0.654 | 0.151 | 0.646 |  |  |
|  | Married/cohabiting | 0.70 (0.42-1.15) | 1.00 (0.86-1.17) | 0.88 (0.75-1.03) | 0.79 (0.71-0.87) | 0.87 (0.76-0.99) | 0.071 |
|  | Single | 0.85 (0.23-3.12) | 0.75 (0.59-0.96) | 0.85 (0.68-1.05) | 0.72 (0.61-0.86) | 0.76 (0.68-0.86) | 0.698 |
| **Heart diseases** | |  |  |  |  |  |  |
|  | *P* for interaction | 0.226 | 0.216 | 0.154 | 0.957 |  |  |
|  | Married/cohabiting | 0.97 (0.66-1.43) | 1.02 (0.87-1.19) | 1.01 (0.87-1.18) | 0.89 (0.82-0.97) | 0.95 (0.87-1.04) | 0.317 |
|  | Single | - | 1.15 (0.93-1.41) | 1.20 (0.98-1.46) | 0.78 (0.68-0.90) | 1.02 (0.77-1.34) | <0.001 |
| **Stroke** | |  |  |  |  |  |  |
|  | *P* for interaction | 0.980 | 0.638 | 0.765 | 0.083 |  |  |
|  | Married/cohabiting | 0.69 (0.36-1.30) | 0.97 (0.68-1.38) | 0.86 (0.69-1.06) | 0.77 (0.67-0.88) | 0.81 (0.72-0.90) | 0.560 |
|  | Single | 0.92 (0.15-5.85) | 1.02 (0.66-1.56) | 0.82 (0.60-1.12) | 0.70 (0.57-0.87) | 0.79 (0.65-0.95) | 0.455 |

Notes: CMDs, cardiometabolic diseases. CMM, cardiometabolic multimorbidity. CHARLS, China Health and Retirement Longitudinal Study. MHAS, Mexican Health and Aging Study. HRS, Health and Retirement Study. SHARE, Survey of Health, Ageing and Retirement in Europe. sHR, subdistribution hazard ratio. CI, confidence interval. All models were adjusted for age, sex, education, household income, current smoking, current drinking, abnormal weight, and hypertension.
